# Evaluation of Gamma-Aminobutyric Acid (GABA) as a Functional Feed Ingredient on Growth Performance, Immune Enhancement, and Disease Resistance in Olive Flounder (*Paralichthys olivaceus*) under High Stocking Density

**DOI:** 10.3390/antiox13060647

**Published:** 2024-05-25

**Authors:** Jinho Bae, Mohammad Moniruzzaman, Hyeong-Woo Je, Seunghan Lee, Wonsuk Choi, Taesun Min, Kang-Woong Kim, Sungchul C. Bai

**Affiliations:** 1 Aquafeed Research Center, National Institute of Fisheries Science, Pohang 37517, Republic of Korea; jinhobae@korea.kr (J.B.); kangwoongkim@koera.kr (K.-W.K.); 2 Department of Animal Biotechnology, Jeju International Animal Research Center (JIA), Sustainable Agriculture Research Institute (SARI), Jeju National University, Jeju 63243, Republic of Korea; monir1983@jejunu.ac.kr; 3 Department of Fisheries Biology, Pukyong National University, 45, Yongso-ro, Nam-gu, Busan 48513, Republic of Korea; jhw245@naver.com; 4 Department of Aquaculture and Aquatic Science, Kunsan National University, Gunsan 54150, Republic of Korea; lsh@kunsan.ac.kr; 5 CJ Feed & Care, AN R&D Center, 170, Eulji-ro, Jung-gu, Seoul 04548, Republic of Korea; ws.choi14@cj.net; 6 Department of Animal Biotechnology, Bio-Resources Computing Research Center, Sustainable Agriculture Research Institute (SARI), Jeju National University, Jeju 63243, Republic of Korea; tsmin@jejunu.ac.kr; 7 Feeds & Foods Nutrition Research Center, Pukyong National University, Busan 48547, Republic of Korea

**Keywords:** γ-aminobutyric acid, crowding effect, growth performance, innate immunity, disease resistance, olive flounder

## Abstract

Gamma-aminobutyric acid (GABA) is a non-protein amino acid that is found in the brain and central nervous system of animals as an inhibitory neurotransmitter. It has been shown to have a variety of physiological functions, including stress reduction and immune enhancement. This study investigated the effects of dietary supplementation with GABA on growth, serum biochemistry, innate immunity, and disease resistance in juvenile olive flounders (*Paralichthys olivaceus*) challenged with *Edwardsiella tarda* under high-stocking density. A control diet and three experimental diets were prepared, with 150 mg/kg (GABA150), 200 mg/kg (GABA200), and 250 mg/kg (GABA250) of GABA added to each diet, respectively. Each experimental diet was fed to olive flounders in triplicate with an initial weight of 12.75 g ± 0.3 g in 40 L tanks at two stocking densities: normal density (20 fish/tank) and high density (40 fish/tank). After 8 weeks of the feeding trial, growth, feed utilization, whole-body proximate compositions, blood analyses, and non-specific immune responses were measured, and challenge tests were performed. There were no significant differences in the weight gain (WG) and specific growth rate (SGR) among fish fed the GABA-supplemented diets at the two stocking densities. However, the normal-density groups showed significantly higher WG and SGR than the high-density groups (*p* < 0.05). There was no significant difference in feed efficiency and protein efficiency ratio among all groups. Moreover, there was no significant difference in the whole-body proximate composition analysis (*p* > 0.05). There were no significant differences in cortisol levels in fish fed the GABA at both densities, but the high-density group showed a significantly higher cortisol than the low-density group. Blood GABA significantly increased in a dose-dependent manner regardless of the density groups (*p* < 0.05). Superoxide dismutase activity showed significantly higher levels than the control group, but there was no significant effect of the stocking densities in fish fed the GABA diets (*p* < 0.05). Myeloperoxidase activities in fish fed the GABA200 and GABA250 diets showed significantly higher levels at both of the stocking densities (*p* < 0.05). Lysozyme activity was significantly higher in the GABA150 group than in the CON, GABA200, and GABA250 groups (*p* < 0.05). After 15 days of challenge tests with *Edwardsiella tarda*, the cumulative survival rates of the GABA150, GABA200, and GABA250 groups were significantly higher than that of the CON group (*p* < 0.05). The results suggested that the optimal dietary GABA level for juvenile olive flounder culture is 150 mg/kg, regardless of rearing density, to enhance growth, immunity, and disease resistance.

## 1. Introduction

The world population is exploding, and food crises continue to emerge. Accordingly, many scientists and researchers in all fields, such as agriculture, livestock, and fisheries, are working hard to solve the food crises that will come. Among them, aquaculture is an emerging industry for food security and plays a very important role as a protein source. In fact, aquaculture production has increased by 7 percent per year over the past 30 years (CAGR: compound annual growth rate), and in 2018, it produced 115 million tons, and this trend is expected to continue in the future. On the other hand, fishery production has stagnated at about 98 million tons from 2004 to the present [1]. The aquaculture industry is developing radically due to the standstill of fishery production and the increasing demand for protein and aquatic products in modern society. The aquaculture industry has been changing from a labor-intensive primary industry to a knowledge-intensive fourth industry that utilizes Big Data, AI, and ICT [2,3]. What is important in this change is the stocking density of fish. As the aquaculture industry develops, high-density culture occurs, and the stress of fish increases due to high-density culture, which increases the fish’s disease susceptibility and eventually leads to mass death [4]. For a successful aquaculture industry, the development of ways that can reduce the stress level of fish is essential. However, in the current aquaculture industry, diseases are suppressed by using large amounts of antibiotics, not substances that lower the stress level of fish. Due to the excessive use of these antibiotics, many pathogens have recently become resistant to antibiotics, and in particular, bacteria resistant to multiple antibiotics have emerged [5]. In addition, when antibiotics are added to feed, the feeding environment, and eventually aquatic products, they remain or accumulate in the water, damaging the health of end-users and humans and polluting the aquatic environment due to antibiotics flowing into the natural environment. Therefore, research on antibiotic substitutes and alternatives that can lower the stress level is indispensable.

Gamma-aminobutyric acid (GABA), used in this experiment, is a natural substance present in bacteria, plants, and animals and is synthesized from glutamic acid inside the presynaptic synapse of inhibitory nerve cells [6,7,8]. Glutamic acid decarboxylase (GAD) makes GABA by removing carboxyl groups from glutamic acid. Although it is a kind of amino acid in terms of molecular structure, it is not used for protein synthesis [9,10,11]. Its chemical formula is C_4_H_9_NO_2,_ and its mass is 103.120 g/M. The synthesized GABA is stored in synaptic vesicles, released to the synaptic cleft, and binds to the GABA receptor (GABA_A_ receptor & GABA_B_ receptor) located on the post-synaptic surface to activate the receptor [12]. The GABA_A_ receptor is a ligand-gated ion channel that passes chloride ions when GABA binds. In the developed brain, the concentration of chloride ions outside neurons is higher than the inside, so when the GABA_A_ receptor opens, chloride ions enter the neurons, which induces the hyperpolarization of neurons, suppressing excitability and suppressing the occurrence of action potentials [13,14]. The GABA_B_ receptor is a G protein-associated receptor that does not directly act as an ion channel, but it inhibits the excitability of nerve cells by indirectly controlling various ion channels, such as potassium ion channels, through G protein signaling. When there is a problem in the production of glutamic acid decarboxylase (GAD) and GABA synthesis, the amount of GABA in the brain decreases, which causes a decrease in the activity of inhibitory synapses. Neurons that are not normally inhibited are overexcited, which in turn leads to abnormal neuronal activity [15]. In mammals, when there is a problem with GABA synthesis, it has been reported that schizophrenia and bipolar disorder occur [16,17,18,19].

GABA induces stable metabolic processes and immune and antioxidant responses by inhibiting overactive neurons in stressful situations. Because of this effect, it is a feed additive used in the poultry industry and livestock industry for various purposes, such as stress reduction, immunity enhancement, antioxidant status, and the promotion of feeding [20,21,22]. Although it is a feed additive that has not been largely researched in fish, studies on feed with GABA are also being conducted in aquaculture diets. According to previous research works [23,24,25,26,27,28,29], GABA has also been proven to have a positive effect on fish and shrimp. In juvenile grass carp, 87.5 mg/kg of GABA in feed had a positive effect on growth and antioxidant ability [24]. Likewise, dietary GABA at 158.7 mg/kg in Nile tilapia showed an effect on growth, feed utilization ability, and homeostasis ability [25]. In whiteleg shrimp, 150 mg/kg of GABA showed an effect on growth and antioxidant capacity [26]. However, there is a scarcity of research studies under any stress conditions, especially the evaluation of dietary supplementation of GABA in fish under high stocking density stress.

Olive flounder is one of the most consumed and commercially important marine demersal fish species cultured in East Asian countries like the Republic of Korea, Japan, and China [27,28]. Recently, a series of experiments have been conducted by our group [25,27,28,29] where we documented that dietary GABA had a beneficial effect on growth performance, feed utilization, hematology, innate immunity, and disease resistance in juvenile olive flounder, Nile tilapia, and whiteleg shrimp. In particular, we have demonstrated that dietary GABA at 200 mg/kg can ameliorate the high-temperature stress in olive flounder [28]. Therefore, in this study, we envisaged to evaluate the growth performance, feed utilization ability, hematological analysis, non-specific immune response, and disease resistance that occurs during normal and high-density culture by adding different concentrations of GABA in feed for olive flounders, *Paralichthys olivaceus*, as a major domestic fish species in the Republic of Korea.

## 2. Materials and Methods

### 2.1. Experimental Design and Diets

Anchovy fish meal, soybean meal, squid liver meal, meat and bone meal, and poultry by-products were used as the main protein sources for the experimental feed. Marine fish oil was used as a lipid source, wheat flour, and cellulose were used for the shape of the feed, and sufficient minerals and vitamins were supplied [30]. For this, a commercial premix was added. Choline, taurine, betaine, lecithin, and monocalcium phosphate were used as other additives, and the composition of the feed source is shown in Table 1. All feed sources were put in a mixer (HYVM-1214, Hanyoung Food Machinery, Hanam-si, Republic of Korea) and mixed well. The feed was prepared using a pellet machine (SMC-12, SUN Engineering, Seoul, Republic of Korea), dried at room temperature for 48 h, and stored in a freezer at −20 °C. GABA was used in powder form obtained from Mirae Resources company (Seoul, Republic of Korea) and showed a purity of 76.5% GABA as a result of analysis with high-performance liquid chromatography (HPLC). In order to evenly add GABA, which is a very small amount of the experimental feed, it was mixed with cellulose for 72 h using a ball mill machine (PL-BM5L, POONG LIM Trading., Co. Pusan, Republic of Korea) to make cellulose containing 40,000 mg/kg of GABA. After that, four experimental diets were made by adding 0 (CON), 150 (GABA_150_), 200 (GABA_200_), and 250 (GABA_250_) mg of GABA per kg of diet. The HPLC analysis showed that there was 63.92, 231.30, 291.59, and 323.90 mg/kg of actual GABA content in CON, GABA_150_, GABA_200_, and GABA_250_ diets, respectively, as shown in Table 2 and Table 3.

### 2.2. Experimental Fish and Feeding Trial

The olive flounders used in this experiment were transported from the SAMWOO Aqua hatchery (Boryeong, Chungcheongnam-do, Republic of Korea) to the Laboratory of Feeds and Foods Nutrition Research Center (FFNRC) at Pukyong National University. Before starting the experiment, the acclimation phase was carried out by mixing and supplying commercial feed and CON for 3 weeks in a 2000 L tank. After the acclimation phase, olive flounders with an average weight of 12.75 ± 0.5 g were reared in each of the 40 L square tanks containing 20 fish as the normal density, or ND (240 fish), and 40 fish as the high density, or HD (480 fish), in 24 tanks (total 720 fish) with triplicate groups (one treatment consisting of three replicate tanks) based on the dietary treatments. Each experimental tank was controlled so that the amount of water flow was 2.0 L/min by a semi-recirculating system, and 150% of the water was exchanged every day. The water temperature was set to 17.5 ± 1 °C, and the salinity of the water was maintained at 33 ± 1 ppt. For sufficient oxygen supply, an air stone was installed in each tank to keep the dissolved oxygen content at 6–7 ppm, and the feed was supplied by adding 3–4% of the fish’s body weight twice a day.

### 2.3. Sample Collection and Analyses

#### 2.3.1. Growth Performance

After the 8-week culture experiment was over, the total weight of the fish in each tank was measured after fasting for 24 h in order to investigate the growth performance and feed efficiencies. The equations for the WG, FE, SGR, and PER analyses are as follows.

Weight gain (WG, %) = [final weight − initial weight] × 100/initial weight

Feed Efficiency (FE, %) = [wet weight gain/dry feed intake] × 100

Specific growth rate (SGR, %/day) = [loge final weight − loge initial weight] × 100/days

Protein efficiency Ratio (PER) = Wet weight gain/Protein intake

#### 2.3.2. Proximate Analysis

Proximate analysis was performed according to the standard AOAC [31] method. Moisture and ash were measured by placing a sample of a constant weight for 24 h in a dry oven at 105 °C and burning it in a muffle furnace at 500 °C for 3 h. For the analysis of the crude lipid and crude protein content of feed and whole fish, the sample was frozen at −20 °C for 24 h and then made into a powder form using a freeze-drying machine (Advantage 2.0, VirTis, New York, NY, USA). Afterward, the crude protein was analyzed using the Auto Kjeldahl system (Buchi B-324/435/412, Switzerland; Metrohm 8-719/806, Switzerland) by Kjeldahl nitrogen determination (Nitrogen × 6.25). (Tecator AB, Hoganas, Sweden). In addition, the actual GABA content of the experimental feed was analyzed using HPLC at the National Instrumentation Center for Environmental Management, College of Agriculture and Life Science at Seoul National University (Seoul 151-742, Republic of Korea).

#### 2.3.3. Hematological Parameters

After the eight-week feeding trial, 6 experimental fish were randomly selected from each tank, and blood was collected from the caudal vein using a non-heparinized 1 mL syringe. In order to analyze the amount of GABA remaining in the blood, the blood was collected 24 h after feeding. The blood was centrifuged (5000× *g*) for 10 min, and the serum was retaken and immediately placed at −70 °C. Glutamic oxaloacetic transaminase (GOT), Glutamic pyruvic transaminase (GPT), and Glucose from Fuji DRI-CHEM 3500i (Fuji Photo Film Ltd., Tokyo, Japan) was used.

A cortisol ELISA kit (ENZO Life Sciences Inc., New York, NY, USA; ADI-900-071) was used to analyze cortisol in the blood and carried out according to the manufacturer’s standard protocol. The kit for the quantitative measurement of cortisol uses a monoclonal antibody to cortisol to bind, in a competitive manner, cortisol or an alkaline phosphatase molecule that has cortisol covalently attached to it in a sample. After a simultaneous incubation at room temperature, the excess reagents were washed away, and substrate was added. After a short incubation time, the enzyme reaction was stopped and read on a microplate reader at 405 nm. The measured optical density was used to calculate the concentration of cortisol.

To analyze the GABA in the blood, a QuickDetect™ GABA ELISA Kit (Biovision Inc., San Francisco, CA, USA; E4455-100) was used. The analysis method was carried out according to the manufacturer’s standard protocol. In addition, the blood was collected and analyzed exactly 24 h after feeding to determine the amount of GABA in the blood.

#### 2.3.4. Non-Specific Immune Response

Lysozyme activity was measured using the absorbance from the microplate technique. For this measurement, a bacterial suspension was prepared by mixing 0.2 mg of Micrococcus lysodeikticus (Sigma, Perry, GA, USA) to each sample in 1 mL of sodium citrate buffer (0.02 M, pH 5.52). A serum dilution was prepared by mixing 30 μL of serum and 270 μL of sodium citrate buffer (0.02 M, pH 5.52). A 180 μL bacterial suspension was placed into each 96-well plate, and the 20 μL serum dilution was subsequently added and directly incubated thereafter at room temperature for 30 min. The decrease in absorbance was then recorded at 450 nm at 0, 30, and 60 min using a microplate reader (Sunrise TECAN, Männedorf, Switzerland). The active unit of lysozyme was defined as the amount of enzyme showing a decrease in absorbance of 0.001 per minute.

Serum was isolated from fish in each experimental section. Using the SOD assay kit (Sigma-Aldrich, St. Louis, MI, USA, 191600), according to the manufacturer’s instructions, the inhibition rate of the enzyme was calculated as a percentile with WST-1 (Water soluble tetrazolium dye) and xanthine oxidase. Each sample was reacted in a 37 °C incubator for 20 min, and then absorbance was measured at a wavelength of 450 nm (absorbance of the wavelength to measure the color displayed by the reaction of WST-1 and reactive oxygen) using a microplate reader (Sunrise TECAN, Männedorf, Switzerland). The inhibition rate was expressed in units of SOD activity per mg protein.

The measurement of myeloperoxidase (MPO) activity was determined broadly with 20 μL of serum that was diluted with 80 μL of HBSS (Hanks Balanced Salt Solution), without Ca^2+^ or Mg^2+^, in 96-well plates. A total of 35 μL of 3, 3′, 5, 5′ tetramethylbenzidine hydrochloride (TMB, 20 mM) (Sigma Aldrich) and H_2_O_2_ (5 mM) was added. After the addition of 35 μL of 4 M sulfuric acid, the absorbance was measured at a 450 nm wavelength after a 2 min incubation in a 37 °C incubator.

#### 2.3.5. Challenge Test

In the challenge test, *Edwardsiella tardar* was incubated at 27 °C for 24 to 48 h in BHI broth medium and then suspended in sterile distilled water at 1 × 10^7^ CFU/mL. After injecting 0.1 mL of the suspension into the peritoneal cavity of 15 fish randomly selected from each experimental feed, the survival rate of the olive flounders was investigated according to the elapsed time without feeding; the experimental group and the control group were compared and analyzed.

### 2.4. Statistical Analysis

The arrangement of the experimental tank was completely random, and the growth and analysis results were statistically analyzed by two-way ANOVA using the IBM SPSS 26 statistics program. When a significant difference was observed, a Least Significant Difference (LSD) test was used to compare means. Treatment effects were considered significant at *p* < 0.05.

## 3. Results

### 3.1. Interactive Effects of GABA and Stocking Density on Growth Performance of Olive Flounder

The growth performances of the juvenile olive flounders fed different experimental diets supplemented with GABA at low and high stocking densities are presented in Table 4. After 8 weeks of the feeding trial, the weight gain (WG, %), specific growth rate (SGR, %/day), feed efficiency (FE, %), and protein efficiency ratio (PER) were measured. As a result, there were no significant differences according to the GABA level. In addition, there was no significant interaction effect between the GABA level and the density in all experimental groups. However, the normal-density group showed significantly higher effects than the high-density group in terms of WG and SGR (*p* < 0.05).

### 3.2. Interactive Effects of GABA and Stocking Density on Proximate Composition of Olive Flounder

After 8 weeks of the feeding trial, there were no significant differences between the GABA levels and densities in all experimental groups. There were also no significant interaction effects between the GABA level and density (*p* < 0.05) among fish fed the GABA diets at the two stocking densities. The results of the whole-body proximate composition of fish are presented in Table 5.

### 3.3. Interactive Effects of GABA and Stocking Density on Hematology of Olive Flounders

The results of the hematological parameters are presented in Table 6. At the end of the feeding trial, glutamic oxaloacetic transaminase (GOT), glutamic pyruvate transaminase (GPT), and glucose (GLU) were not significantly affected by the dietary GABA level and density in all experimental groups. Also, there was no significant interaction effect between the GABA level and density (*p* > 0.05). However, there was a significant effect of densities on fish fed the experimental diets.

### 3.4. Interactive Effects of GABA and Stocking Density on Immune Response in Olive Flounder

The results of the present study related to immune response are reflected in Table 7. After 8 weeks of the feeding trial, there were no significant density or interaction effects between the GABA level and density in terms of lysozyme activity, myeloperoxidase activity (MPO, absorbance), and superoxide dismutase (SOD, % inhibition) in fish fed the GABA diets in the two stocking densities (*p* > 0.05). However, there were significant effects of the GABA level on SOD, MPO, and lysozyme levels. The lysozyme activity of fish fed GABA_150_ was significantly higher than those of fish fed the CON, GABA_200_, and GABA_250_ diets at both stocking densities (*p* > 0.05). However, the MPO levels in fish fed the GABA_200_ and GABA_250_ were significantly higher than those of fish fed the CON and GABA_150_ diets (*p* < 0.05). The SOD activities in fish fed the GABA_250_ and GABA_150_ diets were significantly higher than those of fish fed the CON diet, and there were no significant differences between fish fed the GABA_150_, GABA_200_, and GABA_250_ diets at normal or high stocking densities (*p* > 0.05).

### 3.5. Interactive Effects of GABA and Stocking Density on Challenge Test in Olive Flounder

**Figure 1:**
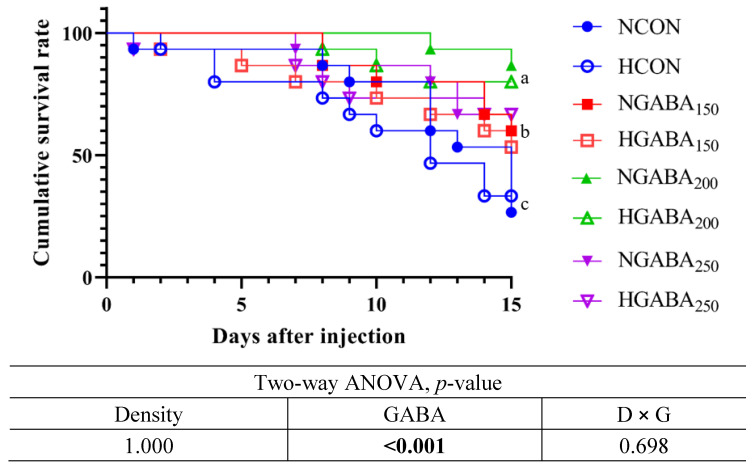
Challenge test with *Edwardsiella tarda* (1 × 10^7^ CFU/mL) ^1^. ^1^ Cumulative survival percentage of olive flounders fed four different experimental diets and two different stocking densities (*n* = 3) for 8 weeks and challenged with *Edwardsiella tarda* (1 × 10^7^ CFU/mL) for 15 days. NCON: Normal Density + CON diet; HCON: High Density + CON diet; NGABA_150_: Normal Density + GABA_150;_ HGABA_150_: High Density + GABA_150_; NGABA_200_: Normal Density + GABA_200_; HGABA_200_: High Density + GABA_200_; NGABA_250_: Normal Density + GABA_250_; HGABA_250_: High Density + GABA_250_.

After 8 weeks of the feeding trial, the challenge test with pathogenic bacteria (*Edwardsiella tarda* 1 × 10^7^ CFU/mL) for 15 days showed a significantly higher cumulative survival rate for fish fed the GABA_150_, GABA_200_, and GABA_250_ diets compared to fish fed the CON diet at both stocking densities (*p* < 0.05). However, there were no significant effects of density or an interaction between density and GABA observed in terms of cumulative survival in fish fed the GABA-supplemented diets at normal or high stocking densities. The results refer to Figure 1.

## 4. Discussion

GABA, with four carbon non-protein amino acids, can be used as a safe feed additive [32], which may play a pivotal role as a primary inhibitory neurotransmitter [33] in reducing neuronal excitability throughout the nervous system and alleviating the intensity of stress in organisms [34]. Moreover, GABA has been known to possess antidiabetic, antioxidant, and immune-modulating properties [35]. Due to the well-reported biological activities, GABA as a functional feed additive has been widely used in the animal industry to improve growth performance and to prevent heat stress-related signs in farm animals [36], fish, and shrimp [23,24,25,26,27,28,29]. The results of this experiment showed that dietary GABA did not reduce the stocking density stress. Therefore, the WG and SGR results were significantly higher in the normal density group. Compared with previous experiments conducted on olive flounders in our laboratory [27,28], there was a significant difference in growth performance at a specific GABA level, and the same results were obtained for other fish species such as Nile tilapia, grass carp, and whiteleg shrimp [24,25,26]. These showed results that were contrary to those of our current experiment. However, in agreement with the present study, Jeong et al. [32] found that dietary GABA did not influence growth performance, and no interaction between stocking density and GABA was noted in chickens. Furthermore, in this study, feed utilization, in terms of FE and PER, was reduced at a high stocking density of fish regardless of the dietary supplementation of GABA in the diets, which is consistent with the feed data found in chickens by Jeong et al. [32]. The results might be due to the stocking density being too high compared to the carrying capacity that the culture system in the laboratory can handle, which is considered to have influenced the normal density group as well. Therefore, if designing such an experiment in the future, another culture system should be introduced to minimize the influence between density groups. There was no significant difference in feed utilization abilities such as FE and PER. This was probably due to GABA being converted by GABA transaminase and glutamine synthetase into glutamate and glutamine, which affects the tricarboxylic acid (TCA) cycle and affects the glial cells that provide energy to neurons, thereby reducing glucose oxidation in oxidative metabolism. Because it reduces glucose oxidation, it affects energy metabolism [36,37,38,39]. Therefore, it is considered that there were no significant differences in feed utilization. In addition, we could see the effect of GABA in tilapia, white shrimp, and grass carp with the same results [24,25,26]. However, the exact pathways of inhibitory neurotransmitters for energy metabolism have not yet been identified [24,25,26]. The lack of observed effects on growth performance and feed utilization in our study may not solely stem from the levels of dietary GABA used. Other factors, such as environmental stressors and the complex interplay of variables in aquaculture systems, could have influenced the outcomes. For instance, while GABA may have demonstrated efficacy in mitigating growth depression induced by heat stress in a previous study [28], its effectiveness in addressing growth depression due to stocking density remains uncertain. Further research is warranted to better understand the nuanced effects of dietary GABA in different aquaculture conditions.

Hematological analysis is an important indicator of the health and physiological condition of fish [40]. GOT (=aspartate aminotransferase) and GPT (=alanine aminotransferase) are enzymes present in hepatocytes and are mainly released into the blood when hepatocytes are damaged. As a result of the GOT and GPT analyses, there were no significant differences between all experimental groups, which is a good indicator of the beneficial effects of GABA as a feed additive [41,42]. Likewise, blood GLU levels were not significantly affected by the GABA level, density, or their interaction. In addition, blood cortisol levels were not significantly affected by the GABA level and the interaction effect of GABA and density. However, the high-density group showed significantly higher cortisol levels than the normal-density group (*p* < 0.05). Blood GABA content was significantly increased according to GABA intake without density and interaction effects (*p* < 0.05). The analysis of GABA content in blood to determine the amount of GABA actually digested after ingestion showed a result that was significantly increased with the amount consumed, irrespective of the density groups. This means dietary GABA is well absorbed in olive flounders [43]. The content of cortisol in the blood is a representative stress indicator [44]. In cases of chronic stress, the inter-renal cells of the kidney head are stimulated through the HPI axis to promote cortisol secretion, and metabolic reactions are promoted, thereby increasing the concentration of glucose in the blood. In the present study, the high-density group showed significantly higher cortisol levels compared to the normal-density group, which confirmed that dietary GABA did not show a direct effect on reducing stocking density stress. However, blood cortisol levels in fish fed the GABA diets with the same stocking densities (normal or high) and GLU levels in both stocking densities did not significantly differ, which is in agreement with the results found in *Labeo rohita* fingerlings [44]. In contrast to the present study, fish, broiler chickens, Roman hens, pigs, and cows showed the effect of reduced temperature stress with GABA supplementation [21,28,45,46,47]. This means that dietary GABA plays an important role in temperature stress rather than density stress, and it is necessary to proceed with a follow-up study. In addition, glucose levels were not significantly different between all experimental groups, which is believed to be maintained at a similar level of blood glucose by insulin, thyroid hormone, and other hormones that control blood glucose levels in the body [48,49]. In agreement with the present study, Jeong et al. [32] reported that blood GPT, GOT, and GLU levels in chickens under different stocking densities were not significantly affected when animals were fed GABA-supplemented diets. Likewise, the researchers also found that GABA had no effect in reducing corticosterone levels (stress indicator) in chickens raised in a high stocking density, which also endorsed the findings of the current study.

A non-specific immune response is the innate immune system that reacts primarily in the body when exposed to dangerous substances. The reaction to external substances or abnormal substances (infectious substances or chemical/physical damage) occurs immediately and non-selectively, thus acting as an important indicator of the immune response [50,51]. SOD is an enzyme that acts as an antioxidant defense mechanism in almost all cells exposed to oxygen. As a result of the analysis of the current study, significantly higher SOD levels were obtained in the experimental groups to which GABA was added. It was found that GABA decreased oxidative stress and increased the activity of antioxidant enzymes in fish, as found in humans, rats, broilers, hens, and pigs, as well as similar to those found in fish and shellfish [15,20,21,22,23,24,25,26,27,28,29,45]. The results of the present study indicate that dietary GABA can attenuate stress in fish due to high stocking density, which is in agreement with results found in the case of the antioxidant enzyme SOD in Roman hens exposed to heat stress [44], the glutathione (GSH) enzyme in largemouth bass fish exposed to ammonia stress and in turbot fed a high soybean meal diet [52,53]. MPO is an enzyme mainly contained in neutrophils. It catalyzes the reaction to produce hypochlorous acid (HOCl) from hydrogen peroxide (H_2_O_2_) and chloride ions (Cl^−^) and has antibacterial and antiviral effects. In the present study, significantly higher MPO levels were found in fish fed the GABA_200_ and GABA_250_ diets under normal and high stocking densities, which is consistent with the results related to malondialdehyde (MDA) and nitric oxide (NO) levels in largemouth bass fish exposed to ammonia stress and in turbot fed a high soybean meal diet [52,53]. A lysozyme is an enzyme that destroys the cell wall of bacteria and plays an important part in the innate immune system. In this study, fish fed GABA_150_ had significantly higher levels of lysozymes compared to the other groups in the two stocking densities. Ruenkoed et al. [54] reported that the dietary supplementation of GABA in Nile tilapia, in terms of antioxidant activities such as SOD and glutathione peroxidase (GPx), were unaffected under ammonia stress which supported the data of SOD and lysozyme enzymes in the current study. The results of the present study confirmed that treatment with GABA or GABAergic drugs is related to improved macrophage maturation, autophagy activation, and antibacterial response to bacterial infection and enhanced the host’s innate immune response [55]. In summary, it can be corroborated that the innate immune response was enhanced in the experimental groups to which GABA was added regardless of the rearing density. However, different trends were shown depending on the amount of GABA added to the feed, so the amount of GABA added according to the fish species and size should be identified. However, there is no fully established mechanism for the interaction between the nervous and immune systems. In particular, it has not been studied mainly in fish. However, according to recently published articles, the effect of enhancing immunity through macrophages and autophagy using in vivo GABA and GABAergic is gradually revealed [56,57,58]. At the end of the 15-day challenge test with pathogenic bacteria, *E. tarda*, dietary GABA showed a high cumulative survival rate in fish under normal and high stocking densities, which is consistent with the previous studies related to GABA supplementation in fish and shrimp [27,29]. The cost of the GABA used in this study was USD 25/kg, which is comparable to other feed additives used in the global aquaculture industry. However, further economic feasibility analysis can be conducted during the dissemination of this technology at the farm level.

## 5. Conclusions

Rearing density has always been an important topic in the aquaculture industry. Efforts to create high production per area are ongoing, and various aquaculture technologies are being developed. However, the high stocking density increases the risk of mass mortality because as the stress on fish increases, low growth performance and disease susceptibility increase. In order to prevent this, it is necessary to develop feed additives to reduce stress and promote immune responses and disease resistance. The gamma-aminobutyric acid used in this experiment is an inhibitory neurotransmitter and is a feed additive already used in livestock industries such as poultry, pig, and cow farming. Therefore, this study was conducted to see the effect of enhancing fish growth and immunity by adding dietary GABA under density stress. These results indicated that dietary gamma-aminobutyric acid (GABA) did not affect olive flounder growth performance but could have a beneficial effect on immune responses and disease resistance. However, the non-specific immune response showed a positive effect according to the different GABA levels. It is necessary to experiment by dividing the GABA level more accurately in future experiments. Lastly, as mentioned in the body of this paper, the gut-brain axis and microbiota is a model that helps to establish explanations for the beneficial effects found not only by GABA but also by other microbial metabolites in the gut of fish. Though this system provides an excellent basis upon which to assert a mechanism for GABA’s systemic effects, the scope of the current trial focuses primarily on GABA’s general effects on growth and immunity that can be assessed by using tests common to short-term feeding trials with juvenile fish. Therefore, in future studies, it is necessary to observe the exact activity of neurons or immune cells and to study other environmental stresses, such as temperature stress.

## Figures and Tables

**Table 1:** Ingredients and formulation of the experimental diets.

	Diets %			
Ingredients	CON	GABA_150_	GABA_200_	GABA_250_
Anchovy FM ^1^	50.00	50.00	50.00	50.00
Soybean meal ^1^	15.00	15.00	15.00	15.00
Wheat flour ^2^	13.70	13.83	13.70	13.58
Squid Liver Powder ^1^	4.00	4.00	4.00	4.00
Meat & Bone meal ^1^	4.00	4.00	4.00	4.00
Poultry by-product ^1^	4.00	4.00	4.00	4.00
Fish oil ^1^	4.00	4.00	4.00	4.00
Lecithin ^1^	0.50	0.50	0.50	0.50
Betaine ^1^	1.00	1.00	1.00	1.00
Taurine ^1^	0.50	0.50	0.50	0.50
Mono calcium phosphate ^3^	0.50	0.50	0.50	0.50
Mineral mix ^4^	1.00	1.00	1.00	1.00
Vitamin mix ^5^	1.00	1.00	1.00	1.00
Choline ^3^	0.30	0.30	0.30	0.30
Cellulose ^1^	0.50			
Cellulose (included 40,000 ppm of GABA) ^6^	0.000	0.375	0.500	0.625
Total	100.00	100.00	100.00	100.00

^1^ The Feed Co., Seoul, Republic of Korea; ^2^ Samhwa Flourmils Co., Seoul, Republic of Korea; ^3^ Sigma-Aldrich Korea Co., Seoul, Republic of Korea; ^4^ Mineral premix (as g/kg premix): Ferrous fumarate, 12.50; Manganese sulfate, 11.25; Dried ferrous sulfate, 20.0; Dried cupric sulfate, 1.25; Cobaltous sulfate, 0.75; Zinc sulfate KVP, 13.75; Cancium iodate, 0.75; Magnesium sulfate, 80.20; Aluminum Hydroxide, 0.75; ^5^ Vitamin premix (as mg/kg premix): A, 1,000,000 IU; D, 200,000 IU; E, 10,000; B1, 2000; B6, 1500; B12, 10; C, 10,000; Calcium pantotenic acid, 5000; Nicotinic acid 4500; B-Biotin 10; Choline chloride, 30,000; Inositol, 5000. ^6^ Milae Bioresources Co., Seoul, Rep Korea; CON: Control; GABA_150_: GABA 150 mg/kg added; GABA_200_: GABA 200 mg/kg added; GABA_250_: GABA 250 mg/kg added.

**Table 2:** Proximate analysis (dry matter basis) of the experimental diets ^1^.

Diets (%)	CON	GABA_150_	GABA_200_	GABA_250_
Moisture	7.65	7.16	7.90	7.95
Protein	52.90	52.10	52.30	52.00
Lipid	10.19	10.20	10.13	10.09
Ash	12.48	12.60	12.42	12.42

^1^ Values are means from triplicate groups (*n* = 3) where the values in each row with different superscripts are significantly different. (*p* < 0.05); CON: Control; GABA_150_: GABA 150 mg/kg added; GABA_200_: GABA 200 mg/kg added; GABA_250_: GABA 250 mg/kg added.

**Table 3:** Actual percentage of GABA according to high-performance liquid chromatography (HPLC) results of the experimental diets.

Diet	CON	GABA_150_	GABA_200_	GABA_250_
GABA (%)	0.006392	0.02313	0.029159	0.03239
GABA (mg/kg)	63.92	231.30	291.59	323.90

GABA: Gamma-aminobutyric acid; CON: Control; GABA_150_: GABA 150 mg/kg added; GABA_200_: GABA 200 mg/kg added; GABA_250_: GABA 250 mg/kg added.

**Table 4:** Effect of dietary gamma-aminobutyric acid (GABA) on growth performance and feed utilization in juvenile olive flounders reared in normal and high stocking densities after 8 weeks ^1^.

Density	ND				HD				Two-Way ANOVA, *p*-Value		
Diet	CON	GABA_150_	GABA_200_	GABA_250_	CON	GABA_150_	GABA_200_	GABA_250_	Density	GABA	D × G
WG ^2^	145.21 ^a^	149.03 ^a^	135.66 ^a^	139.48 ^a^	129.07 ^b^	120.93 ^b^	122.97 ^b^	123.26 ^b^	<0.001	0.292	0.325
SGR ^3^	1.87 ^a^	1.90 ^a^	1.78 ^a^	1.82 ^a^	1.73 ^b^	1.65 ^b^	1.67 ^b^	1.67 ^b^	<0.001	0.302	0.348
FE ^4^	84.10	82.27	73.40 ^a^	81.80 ^a^	77.40 ^a^	73.30 ^a^	70.70 ^a^	77.13 ^a^	0.070	0.213	0.891
PER ^5^	1.59	1.58	1.42	1.57 ^a^	1.46 ^a^	1.41 ^a^	1.37 ^a^	1.48 ^a^	0.067	0.324	0.896

^1^ Values are means from triplicate groups (*n* = 3) of fish where the values in each row with different superscripts (a, b) are significantly different. (*p* < 0.05); ^2^ Weight gain (WG, %) = [final weight − initial weight] × 100/initial weight; ^3^ Specific growth rate (SGR, %/day) = [loge final weight − loge initial weight] × 100/days; ^4^ Feed Efficiency (FE, %) = [wet weight gain/dry feed intake] × 100; ^5^ Protein efficiency Ratio (PER) = Wet weight gain/Protein intake; ND: Normal density; HD: High density; CON: Control; GABA_150_: GABA 150 mg/kg added; GABA_200_: GABA 200 mg/kg added; GABA_250_: GABA 250 mg/kg added.

**Table 5:** Effect of dietary gamma-aminobutyric acid (GABA) on whole-body proximate composition (as is %) of juvenile olive flounders reared in normal and high stocking densities after 8 weeks ^1^.

Density	ND				HD				Two-Way ANOVA, *p*-Value		
Diet	CON	GABA_150_	GABA_200_	GABA_250_	CON	GABA_150_	GABA_200_	GABA_250_	Density	GABA	D × G
Moisture	79.6	80.3	79.7	79.0	79.3	79.1	79.4	79.7	0.154	0.623	0.620
Protein	15.2	14.6	15.2	15.4	15.2	15.6	15.3	14.9	0.064	0.406	0.898
Lipid	1.17	1.18	1.00	1.06	1.16	1.16	1.19	1.13	0.370	0.729	0.177
Ash	3.54	3.84	3.87	3.81	4.04	3.71	3.89	3.89	0.109	0.643	0.623

^1^ Values are means from triplicate groups (*n* = 3) of fish where the values in each row without superscripts are non-significantly different. (*p* < 0.05); ND: Normal density; HD: High density; CON: Control; GABA_150_: GABA 150 mg/kg added; GABA_200_: GABA 200 mg/kg added; GABA_250_: GABA 250 mg/kg added.

**Table 6:** Effect of dietary gamma-aminobutyric acid (GABA) on blood biochemistry of juvenile olive flounders reared in normal and high stocking densities after 8 weeks ^1^.

Density	ND				HD				Two-Way ANOVA, *p*-Value		
Diet	CON	GABA_150_	GABA_200_	GABA_250_	CON	GABA_150_	GABA_200_	GABA_250_	Density	GABA	D × G
GOT ^2^	17.7	18.3	19.0	18.7	16.7 ^a^	16.0	16.7 ^a^	16.0	0.053	0.959	0.936
GPT ^3^	16.7	17.0	16.0	6.3	16.7	15.0	16.3 ^a^	16.0	0.188	0.605	0.149
GLU ^4^	12.7	13.0	11.7	13.3	12.0	14.3	16.0 ^a^	11.7	0.379	0.560	0.145
Cortisol ^5^	2.6 ^b^	1.7 ^b^	1.8 ^b^	1.7 ^b^	4.2 ^a^	5.9 ^a^	5.8 ^a^	5.0 ^a^	0.002	0.980	0.739
GABA ^6^	166 ^a^	220 ^b^	239 ^c^	302 ^d^	175 ^a^	214 ^b^	247 ^c^	293 ^d^	0.790	<0.001	0.059

^1^ Values are means from triplicate groups (*n* = 3) of fish where the values in each row with different superscripts (a, b, c, d) are significantly different. (*p* < 0.05); ^2^ GOT: Glutamic oxaloacetic transaminase (U L^−1^); ^3^ GPT: Glutamic pyruvic transaminase (U L^−1^); ^4^ GLU: Glucose (mg dL^−1^); ^5^ Cortisol: Cortisol in blood (ng mL^−1^); ^6^ GABA: gamma aminobutyric acid in blood (pg mL^−1^); ND: Normal density; HD: High density; CON: Control; GABA_150_: GABA 150 mg/kg added; GABA_200_: GABA 200 mg/kg added; GABA_250_: GABA 250 mg/kg added.

**Table 7:** Effect of dietary gamma-aminobutyric acid (GABA) on non-specific immune responses in juvenile olive flounders reared in normal and high stocking densities after 8 weeks ^1^.

Density	ND				HD				Two-Way ANOVA, *p*-Value		
Diet	CON	GABA_150_	GABA_200_	GABA_250_	CON	GABA_150_	GABA_200_	GABA_250_	Density	GABA	D × G
SOD ^2^	16.24 ^b^	23.15 ^a^	18.78 ^ab^	21.79 ^a^	12.66 ^b^	21.09 ^a^	18.22 ^ab^	22.05 ^a^	0.462	0.046	0.906
MPO ^3^	1.45 ^b^	1.20 ^b^	1.87 ^a^	1.44 ^a^	1.23 ^b^	1.44 ^b^	1.40 ^a^	1.81 ^a^	0.818	0.015	0.006
Lysozyme ^4^	0.15 ^b^	0.39 ^a^	0.10 ^b^	0.25 ^b^	0.17 ^b^	0.39 ^a^	0.26 ^b^	0.14 ^b^	0.542	<0.001	0.520

^1^ Values are means from triplicate groups (*n* = 3) of fish where the values in each row with different superscripts (a, b) are significantly different. (*p* < 0.05); ^2^ SOD: Superoxide dismutase activity (% inhibition); ^3^ MPO: Myeloperoxidase (OD at 450 nm); ^4^ Lysozyme: Lysozyme activity (U mL^−1^); ND: Normal density; HD: High density; CON: Control; GABA_150_: GABA 150 mg/kg added; GABA_200_: GABA 200 mg/kg added; GABA_250_: GABA 250 mg/kg added.

## Data Availability

The original contributions presented in the study are included in the article; further inquiries can be directed to the corresponding authors.
